# What Motivates Participants to Adhere to Green Exercise?

**DOI:** 10.3390/ijerph16101832

**Published:** 2019-05-23

**Authors:** Matthew Fraser, Sarah-Anne Munoz, Sandra MacRury

**Affiliations:** Rural Health and Wellbeing, University of the Highlands and Islands, Inverness IV2 3JH, UK; Sarah-Anne.Munoz@uhi.ac.uk (S.-A.M.); Sandra.MacRury@uhi.ac.uk (S.M.)

**Keywords:** green exercise, motivation, nature, physical activity, sport, adventure sport

## Abstract

There is a lack of research into green exercise which investigates and compares motivational drivers between the different types of outdoor activities. The current paper addressed this gap by classifying and comparing three types of green exercise: (i) Recreational physical activity, (ii) competitive sport, and (iii) outdoor adventure sport. Using a mixed methodological approach, the present study investigated the motivations for adhering to green exercise and directly compared the differences between these three forms of green exercise. Online questionnaires and face-to-face interviews were used to collect data. The results demonstrated that within all types of green exercise, enjoyment was the greatest motivator. Based on analysis of the qualitative materials, extrinsic motivators such as the environment, family, and friends were highlighted as key factors in beginning and continuing their activity. However, intrinsic motivators were also emphasised as more important in adherence to green exercise. Furthermore, as seen in other research, numerous psychological benefits were reported over time. The results of the study may act as a starting point in understanding how we may increase public engagement in green exercise by prompting participants to select a form of green exercise that best suits them based on their motivational profile.

## 1. Introduction

There continues to be a growing concern regarding worldwide decline in physical activity levels [1]. A large proportion of the U.K. population are deemed physically inactive (23% of men and 26% of women) [2]. As a result of this lack of physical activity there is now increased pressure on governments, health care professionals, and academia to identify determinants and correlates of physical activity to improve worldwide health [3]. Previous research has highlighted that individuals should conduct a form of physical activity that they find enjoyable, so as to increase adherence and motivation to meet the recommended physical activity guidelines [4]. The current physical activity guidelines state that individuals should conduct 30 min of moderate to vigorous physical activity on most days of the week [5]. An increasing area of research into physical activity conducted in nature, termed ‘green exercise’, has shown supplementary benefits to mental and physical health in comparison with exercise undertaken in indoor environments [6]. Researchers have predicted that green exercise has the capacity to save the U.K economy a predicted £2.2 billion annually [7].

Due to the numerous health benefits identified from conducting green exercise, researchers believe this knowledge can be used as a powerful extrinsic motivator to increase physical activity levels [8]. Facilitators to exercise such as enjoyment and social experiences appear to be effective in promoting adherence to new exercise programmes [9]. Green exercise studies have shown enjoyment to be correlated with affective responses and future intention to exercise [3]. Moreover, studies have shown enjoyment to be a key factor in visiting natural areas [10,11]. However, whether motives of enjoyment differ between various forms of green exercise, where the focus is not always on the exercise environment has not been fully explored. The outdoor environment offers a functional environment for social interaction as advocated by Ecological Dynamics Theory and provides many affordances not available in controlled indoor environments and urban exercise settings [12]. As a result, it is likely that the motivation to conduct green exercise may differ from other forms of leisure time physical activity due to a range of factors (environment, weather, topography, and affordances offered by the natural environment). From a physiological perspective, studies have shown that exercising outdoors may be perceived by the exerciser as easier than when performed indoors [3]. If an individual believes and experiences that exercising outdoors feels easier, then this may be a useful mechanism in promoting green exercise and adherence to exercise in general.

A recently conducted study by Calogiuri and Elliott (2017) [13] investigated motives within a national survey of Norwegian adults’ physical activity behaviours. The aim of the study was directed towards green exercise and comparing motivational differences between leisure physical activities conducted indoors and green exercise activities, which they highlighted as a research topic which has received little attention and the present study looks to add to. The authors noted that competitive sports are often undertaken in areas that contain natural features and, therefore, should be considered a form of green exercise. Participation in green exercise was linked with higher ‘convenience’, ‘affective benefits’, and ‘long-term health’ motives. The study found that green exercisers were not driven by external body-orientated motives in comparison to sport- and gym-based exercisers. Furthermore, they found this group tended to focus on more extrinsic factors such as natural surroundings. Participants who were driven by affective benefits from physical activity but not the natural environment were more inclined to conduct gym- or sport-based activities to obtain extrinsic benefits. This was one of the first studies to directly compare motivational differences in green exercise settings. The study concluded that based on their findings, sedentary individuals may be more inclined to conduct green exercise in comparison to indoor modalities. Whilst this study was one of the first to compare green exercise motives, it did not consider outdoor adventure sport participants, who also conduct green exercise in highly natural areas which the present study examines.

A review by Pomfret and Bramwell (2016) [14] investigated the motivational decisions and differences for outdoor adventure participants. The authors define this form of green exercise as activities which “*involve land, air and water-based activities, ranging from short, adrenalin-fuelled encounters, such as bungee jumping and wind-surfing, to longer experiences, such as cruise expeditions and mountaineering*” (p. 2). The review highlighted that motives within this form of exercise are varied based on experience, age, and gender. Furthermore, the environment which adventure sport takes place in can be a large motivating factor. Motivational constructs such as flow, edgework, and reversal theory were found to be key elements to conducting this form of exercise. The review noted that there are several research gaps within the outdoor adventure research field such as understanding and comparing motives within this form of activity and profiling motivations.

Caloguiri and Elliott (2017) [13] also highlight that future research into motivation in green exercise should consider whether motives are more intrinsic or extrinsic through the use of psychological theories of motivation. With this considered, the Self-Determination Theory [15] which is frequently used within a sport, exercise, and physical activity context to understand motivation and behaviour was used as an empirical framework to understand green exercise motives. The theory identifies several types of motivation that drive behaviour: Intrinsic, extrinsic, and amotivation. Extrinsic motivation “*refers to the performance of an activity in order to attain some separable outcome and, thus, contrasts with intrinsic motivation, which refers to doing an activity for the inherent satisfaction of the activity itself*.” ([16], p. 71). Researchers note extrinsic forms of motivation can direct behaviour, but cannot predict adherence to behaviour change and is a short term-based solution [15]. The current study looked to examine whether intrinsic and extrinsic motives can explain green exercise participants adherence and whether green exercise may positively influence behaviour change from a public health perspective. There are various modalities of exercise and sport that are conducted in natural environments. Based on previous studies [13,14], we proposed three levels of green exercise: Recreational physical activity, outdoor competitive sport, and outdoor adventure sport. It is likely that differences will exist between these activities due to varying costs, access to locations, required skills, and hidden entry requirements to specific green exercise activities [17]. Understanding what differences and similarities exist between these forms of green exercise may aid in introducing individuals into an appropriate form of green exercise which alienates their current motivational profile.

### 1.1. Rationale

Gascon et al. (2015) [18] notes that not only is it important to understand the types of activities conducted in green spaces but also the motivations behind the decisions to conduct this form of exercise and use these areas. Whilst the environment is considered to be an important factor in terms of motivation in adventure sport, how it may differ across other forms of green exercise requires greater investigation [14]. In a recent review Bamberg, Hitchings and Latham (2018) [19] highlighted the need for more qualitative research in this field. They also noted that now is the time to examine the factors which encourage or discourage participation in different forms of outdoor activities. With current research regarding the natural environment as a potential mechanism to increase motivation, it was deemed necessary to investigate the extent to which the natural environment motivates those currently engaged in green exercise. Furthermore, due to the range of outdoor activities that can be conducted in natural areas, it is likely that there will be varying motives, which when understood, may help introduce individuals to conduct outdoor activities. The current study also looks to build upon previous research [13] and increase knowledge regarding green exercise motivations to ultimately increase the number of individuals conducting this form of physical activity. With worldwide levels of mental and physical health conditions rising and from an economical perspective, increasing the number of people conducting green exercise appears to be of paramount importance.

### 1.2. Aims

The following study aims to first build on the current evidence base in understanding what motivates those currently engaged in green exercise activities to continue to adhere to such modalities. Secondly, to compare motivational differences between the proposed three levels of green exercise, in order to identify the motivational profiles of the various forms of green exercise to aid in promoting this as a long-term form of physical activity.

### 1.3. Hypothesis

1. Differences will exist between groups in terms of intrinsic and extrinsic motivation;
2. Competitive sport outdoor participants will be less focused on the exercise environment and more on competition and achievement;
3. Based on previous research, the social and enjoyment aspects of green exercise will be large factors in whether individuals conduct these activities and adhere to them;
4. The natural environment will be a motivating factor, particularly for recreational and adventure sport participants.

## 2. Materials and Methods

### 2.1. Research Design

A mixed methods study design was used to collect qualitative and quantitative data on the motivations, perceptions, and attitudes of individuals currently engaged in green exercise. Participants completed a motivational questionnaire based on the framework from a previous study [20] to identify extrinsic factors and aspects of the environment participants acknowledged as increasing their motivation to exercise in nature. Furthermore, participants completed the Physical Activity and Leisure Motivation Scale (PALMS) to assess different components of motivation across the modalities of green exercise. The questionnaires were administered via an internet survey/email response. In the second phase of the study, participants from various outdoor activities were interviewed regarding their motivations, perceptions, and attitudes towards green exercise in order to gain an in-depth understanding of motives and characteristics of the different outdoor group participants.

### 2.2. Study Sample

The study sample was estimated through using a priori power calculation using the software G-power version 3.1.9.4 [21] (effect size = 0.25, power = 0.8, alpha level = 0.05). The calculation determined a minimum sample size of 159. The study sampled participants from a range of green exercise activities. A total of 184 participants completed the two motivational questionnaires. Age ranges across the three types of exercise are demonstrated in Table 1. A total of 6 male and female participants (2 from each defined outdoor activity group) volunteered to take part in the interview phase of the study (lacrosse, walking, snowboarding, sailing, golfing, and marathon runner).

### 2.3. Outcome Measures

#### 2.3.1. Physical Activity and Leisure Motivation Scale (PALMS)

In order to assess the motivation of those currently engaged in green exercise, the PALMS questionnaire was administered [22]. The PALMS was developed based on qualitative research which was found to match the theoretical framework, primarily intrinsic-extrinsic motivation as seen in the Self-Determination Theory. The PALMS demonstrated an acceptable factor structure, internal consistency, test-retest reliability, and criterion validity [23]. The results of the study found the scale to have a Cronbach’s alpha coefficient (α = 0.79). This measure demonstrated good internal consistency. Internal consistency for the different PALMS subscales was adequate, ranging from 0.78 to 0.82. When comparing the correlation between each PALMS sub-scale and the previously validated Recreational Exercise Motivation Measure (REMM), results were also found to be high, varying from 0.79 to 0.95. Finally, with regards to test-retest reliability for the questionnaire, sub-scales ranged between 0.78 and 0.94 over a 4-week period. It was also found to be applicable to diverse physical activity contexts.

#### 2.3.2. Motivational Questionnaire

The motivational questionnaire was based on a previous questionnaire from Skar, Odden and Vistad (2008) [20] (Supplementary materials A) who investigated the motivations of mountain bikers in Norway for bicycling in their everyday lives. Of the original 27 items, 7 were included in the current study. The 7 items were selected based on how relatable they were to all forms of green exercise and to avoid overlap with the PALMS questionnaire items. This was deemed a valid and reliable method to investigate outdoor activity motives as opposed to developing a new invalidated questionnaire. The motivational questionnaire was included in an attempt to gain further understanding into the motives closely related to the natural environment and specifically green exercise.

#### 2.3.3. Interviews 

In addition to the two questionnaires, semi-structured interviews were also conducted to gain further insight into the varying motives behind conducting a range of green exercise activities. A semi-structured interview style, with open-ended questions was selected to allow the chance to build rapport, use prompts, and probe to gain in-depth responses [24].

The interview sections were adapted from a previous qualitative study on green exercise [25]. The interviews in the previous study looked at motivations, perceptions, and barriers for members of an outdoor group and thus were used as a framework for the development of the interview sections in the current study. Interview questions were designed around the research question and current theories in the field [12,26,27]. The created semi-structured interview consisted of 15 questions (Supplementary materials B). In specific interviews this was increased due to the need to further probe responses.

### 2.4. Protocol

Following ethical approval from the University (ETH906), emails were sent to 24 local outdoor activity groups obtained from internet and social media pages from the Scottish Highlands (recreational physical activity groups, outdoor sports groups, and adventure sport clubs). Leaders of the groups were contacted via email or their social media page, requesting the opportunity to gain access to issue questionnaires to the members of the group. In the second phase of the study, interviews were conducted with various members belonging to the different green exercise activities.

### 2.5. Data Collection and Analysis

#### 2.5.1. Primary Motives for Outdoor Activity Participation

As the study was interested in understanding the primary motives for conducting outdoor exercise, the sum score (within each factor) was used to calculate the average score for each of the eight motivation factors, which were then placed in rank order, highest (strongly agree) to lowest (strongly disagree). For the motivational nature questionnaire, each participant score was summed and again averaged in order to allow rank order to be analysed.

#### 2.5.2. Differences between Outdoor Groups

Between groups analysis was conducted to assess whether motivational differences existed between the three outdoor conditions. A Kruskal-Wallis one-way ANOVA was used to examine the differences in the ranking of motives for participation across the three different forms of green exercise. The non-parametric Levene’s test was conducted on each variable to assess variance (Adventure *p* = 0.52; Mastery *p* = 0.873; Physical condition *p* = 0.45; Affiliation *p* = 0.55; Psychological condition *p* = 0.11; Enjoyment *p* = 0.95; Competition/Ego *p* = 0.27; Others expectations *p* = 0.19), (I can be in nature *p* = 0.00; I can relax from daily routines *p* = 0.00; I can experience nature *p* = 0.24; I come to places I am especially attached to *p* = 0.51; I can experience changes in nature *p* = 0.03; I can test new equipment and clothing *p* = 0.19; I enjoy the views whilst exercising *p* = 0.00; Exercising outdoors challenges me more *p* = 0.02; I prefer exercising outdoors to indoors *p* = 0.00). When significant differences were found in the non-parametric Levene’s test, this demonstrated that variances were not equal. For items which had an unequal variance, the Brown–Forsythe test was conducted. Effect sizes were calculated and independent between groups, and Kruskal–Wallis post-hoc ranked tests were conducted to identify where the differences between groups existed. Post-hoc Games–Howell tests were conducted on items which displayed unequal variance. Separate correlation analysis was run between the PALMS subscales and the nature questionnaire items. Spearman’s rank order correlation was conducted to identify the strength and direction between the variables. Pearson’s correlation was used for the PALMS subscale items, due to being a mean score for the items being analysed [28]. Finally, median tests were conducted on the nature questionnaire items as these were single Likert scale ordinal data. Chi-squared tests with effect size were also calculated.

#### 2.5.3. Interview Analysis

Interview data was qualitatively analysed using the six-step thematic analysis outlined by Braun and Clarke (2006) [29]. Thematic analysis is a qualitative analysis method which is conducted through identifying, analysing, and reporting themes. Thematic analysis also allows the interpretation of different aspects from collected data and has been demonstrated to be a valid analysis method for applied health and wellbeing research [30]. As the analysis was driven towards answering a predetermined research question, a ‘theoretical’ or deductive thematic analysis was used [31]. A realist approach was adopted in which a unidirectional relationship can be assumed for participant motivations from what has been described [32]. Validity of the coding and theme development was achieved by gaining feedback from two independent reviewers (S.M., S.-A.M.) [33].

The six steps of the conducted thematic analysis are detailed below:

1. Familiarisation with the data;
2. Generating initial codes;
3. Searching for themes;
4. Reviewing themes;
5. Defining and naming themes;
6. Producing the report.

## 3. Results

When analysing the PALMS questionnaire single items for the competitive sport group, it was found that the top three motives for conducting green exercise were ‘Because it makes me happy’ 4.41, ‘Because it is fun’ 4.41, and ‘To be physically fit’ 4.34. For the recreational green exercise group, it was found that the primary motive to conducting this was again ‘Because it makes me happy’ 4.60, followed by ‘To maintain physical health’ 4.56, and ‘To be physically fit’ 4.52. Finally, the adventure sport group ranked ‘Because it makes me happy’ 4.80, ‘Because it’s fun’ 4.68, and ‘Because it’s interesting’ 4.60. It should be noted that there were differences in the number of participants in the recreational participant group which may reduce statistical power of comparisons [34]. Characteristics are displayed below in Table 1.

PALMS Subscales

The 40 items of the PALMS questionnaire are grouped according to the eight factors (Table 2). To obtain a measure of motivational importance, the average score for all 40 items was calculated. When independently examining the PALMS questionnaire it was found that the most important ranked motive was the enjoyment subscale. 

A Kruskal-Wallis one-way ANOVA revealed a statistically significant difference in the ranking of motives for participation across the three green exercise activities. Significant differences between the groups were found for the psychological condition (*p* = 0.03, *r* = 0.74) and the competition ego subscale (*p* = 0.006, *r* = 0.72). Independent Kruskal–Wallis post-hoc tests for the psychological condition factor demonstrated significant differences between competitive and recreational groups (*p* = 0.02, *r* = 0.53) and competitive and adventure (*p* = 0.02, *r* = 0.54). With regards to the competition ego subscale, differences existed again between competitive and recreational groups (*p* = 0.009, *r* = 0.75) and competitive and adventure (*p* = 0.009, *r* = 0.76). The ranking of each individual subscale across the three forms of green exercise are displayed below in Table 3.

With regards to the adapted nature questionnaire, there were a number of significant differences between the different forms of green exercise. The Kruskal–Wallis one way between groups ANOVA revealed significant differences in several items, ‘I can experience the outdoors combined with exercise’ (*p* = 0.00, *η*^2^ = 0.09), ‘I enjoy the views whilst exercising’ (*p* = 0.00, *η*^2^ = 0.16), ‘I come to places I am especially attached to’ (*p* = 0.02, *η*^2^ = 0.03), and ‘exercising outdoors challenges me more’ (*p* = 0.02, *η*^2^ = 0.03). When unequal variance was assumed the Brown–Forsythe test was conducted. Significant differences were found in items, ‘I can be in nature’ (*p* = 0.00 effect), ‘I can relax from daily routines’ (*p* = 0.007 effect), ‘I can experience changes in nature’ (light, dark, sun-rain) (*p* = 0.03 effect), and ‘I prefer exercising outdoors to indoors’ (*p* = 0.00 effect).

As the nature questionnaire was comprised of single Likert scale items, a median test was also conducted to reveal any further differences between the groups. When comparing the median scores, significant differences were observed between items ‘I can be in nature’ χ^2^ (2) = 16.7, *p* < 0.01, Φ = 0.301, ‘I can relax from daily routines’ χ^2^ (2) = 10.97, *p* = 0.04, Φ = 0.24, ‘I come to places I am especially attached to’ χ^2^ (2) = 8.19, *p* = 0.01, Φ = 0.21, ‘I can experience changes in nature’ (light, dark, sun-rain) χ^2^ (2) = 8.68, *p* = 0.01, Φ = 0.21, and ‘exercising outdoors challenges me more’ χ^2^ (2) = 7.91, *p* = 0.01, Φ = 0.20. Differences between each form of green exercise on the nature questionnaire items is outlined below in Table 4. 

Following conducting the Pearson Correlation Test on the PALMS questionnaire subscales, it was found that the greatest relationship existed between ‘Psychological condition’ and ‘Competition/ego’ displaying a statistically significant strong negative correlation r(13) = −0.81, *p* < 0.01. PALMS subscale correlations are displayed below in Table 5.

Regarding the nature questionnaire, following conducting Spearman’s Rank Order Correlation test, the results revealed a strong-moderate statistically significant correlation between the items ‘I can be in nature’ and ‘I can experience the outdoors combined with exercise’ r_S_(13) = 0.66, *p* < 0.01. The nature questionnaire correlation results are displayed below in Table 6.

### 3.1. Qualitative Results

**Figure 1:**
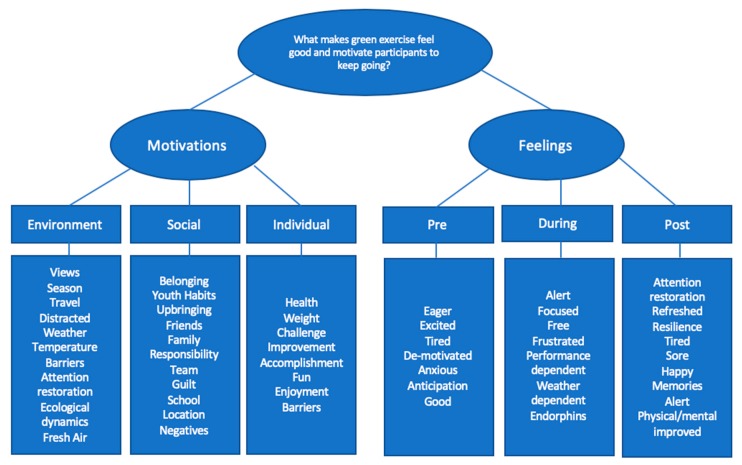
Codes presented as map.

Stage 2 of the study involved interviewing six participants, with a large spectrum of age ranges (25–64 years) from various green exercise activities, data was qualitatively analysed via thematic analysis. Following analysing and coding the collected data, the following themes surfaced via the deductive analysis. In order to provide a greater understanding of the responses in which the themes emerged from, evidence and reasoning for the development of each theme is described below. Figure 1 (below) displays the higher order themes and coding.

In line with the overarching aim and research question of the chapter, two main themes were generated from the qualitative data collection. The main theme of ‘motivation’ was categorised into three sub-themes (1: environment, 2: social, and 3: individual). The second theme of feelings was also categorised into three sub-themes which were based on the time of activity (1: pre, 2: during, and 3: post-exercise).

### 3.2. Motivation

The aim of the qualitative analysis was to understand what motivates those currently engaged in green exercise to continue exercising in natural areas. Secondly, what differences may exist between the different forms of green exercise. From the responses of the six participants it was evident that a mix of the three sub-themes resulted in the participants starting to undertake their activity and continuing to engage in them. Motivation is a highly complex phenomenon and previous research has shown it is made up of several components. An applicable theory to this theme was the Self-Determination Theory (SDT) [15]. The theory states that three elements (autonomy, competence, and relatedness) motivate humans to conduct a behaviour. There are primarily two forms of motivation involved with sport and exercise: External motivation and internal motivation [35]. Both forms of motivation are evident within all forms of the investigated green exercise activities.

#### 3.2.1. Motivation

Sub-theme: Environment

There was a consensus between the different forms of outdoor activities, when questioned on environmental preference all participants agreed that they would opt to conduct outdoor exercise over indoor if given the choice. Participants noted during inclement weather or off seasons, they preferred to not exercise at all than move into an indoor environment. Interestingly, many responses were coded with reference to the Ecological Dynamics Theory [12] in that indoor exercise was seen to be de-motivating and was viewed as a negative due to temperatures and low dynamic sources of information. The Ecological Dynamics Framework highlights that indoor environments provide reduced affordances and as a result, individuals experience decreased psychological responses which was reflected in the participants responses.

Participant 4: “*Definitely outdoors, I don’t really see the interest in running indoors, it is a lack of motivation wise, at least when you are running outside there are more things to see, more things to watch and kind of distract you more than when you’re inside it is more a case of just running for the sake of running on the same spot.*” [Marathon Runner]

Participant 2: “*Outdoors, because indoors it is hot and sweaty and there’s people around you and it is just nicer outside, you feel better.*” [Recreational walker]

Participant 5: “*I don’t see the idea of going inside to snowboard or artificial, nowhere near the same aspect of doing it in real life. I would rather pay the money and get abroad and do it at times of the year when you can’t do it back home.*” [Snowboarder]

In terms of the outdoor environment, participants’ responses all suggested that they would select to exercise in greenspaces in comparison to man-made environments. Responses were closely linked to the Attention Restoration Theory [27], in that participants highlighted aspects of natural environments that distracted or grabbed their attention. Previous studies have shown that when distracted by external stimuli, participants may perceive exercise as easier in natural environments [3].

Participant 2: “*Just because you feel better, it is less noisy, you know you just, it’s just nicer and it is healthier, I don’t know it’s more fresh air.*” [Recreational walker]

Participant 4: “*Well yeah definitely a greenspace I obviously start in the city, so it is a lot more difficult when you are in the city, you’ve got different obstacles you’ve got to move around, which is sometimes good and it takes your mind off actually running. But so does when you’re actually going out to the countryside, you get to see more different views, different scenery.*” [Marathon runner]

Sub-theme: Social

It was evident from the transcribed data that social circles such as friends and family are responsible at some level for introducing an individual to green exercise. It also appears that the location of a person’s upbringing is a factor in whether an individual begins to conduct regular green exercise. The majority of participants noted that having been introduced into their activity by friends and family, they now have continued to take part in their activity for several years. Referring to the Self-Determination Theory [15], it appears that relatedness may be an important factor in motivating participants to undertake outdoor exercise.

Participant 3: “*Started playing golf, maybe about 6–7 years ago now, I got into it because I was a bit older and it was a more sociable game and a lot of my friends were playing it, so I decided to start taking it up.*” [Golfer]

Participant 6: “*I think probably started doing it because I stayed in Findhorn, which is next to the sea and everybody sailed, and my family sailed so I took up sailing at an early age.*” [Sailor]

It was found that the different types of outdoor activities may change the desired social aspect. In contrast to the hypothesis, some participants revealed mixed feelings regarding the social aspects of green exercise. It was clear that teams or groups can promote responsibility, fun, and belonging which results in adherence to exercise and increased levels of enjoyment. This however is not optimal for all those conducting green exercise. The sub-theme of ‘social’ motives displayed contrasting responses between the outdoor activities.

Participant 1: “*When you’re in a team you obviously don’t want to let your team mates down and this is what motivates you to do the best you can, so you bring your a-game.*” [Lacrosse player]

Participant 5: “*I’ve only ever been up once and done it myself. It’s something that I see as needing two people to do it just makes it a bit more enjoyable.*” [Snowboarder]

Participant 2: “*I prefer doing it alone.*” [Recreational walker]

Researcher: “*Any reasons why?*”

Participant 2: “*Just because you can sort of lose yourself and you can go at your own pace and you can go where you want, and you can just please yourself.*”

Participant 4: “*Individually I think it is a lot easier for me, I work a lot better individually, I don’t know if it would alter my performance if it was done in a team but individually is what I choose to do.*” [Marathon runner]

Participant 6: “*You can’t blame anyone else for your mistakes and the good thing about doing it on your own is that you don’t have to rely on anyone to crew with you and crews are quite hard to get so you can just sail when you want.*” [Sailor]

Sub-theme: Individual

Due to the variety of green exercise participants interviewed, each activity had unique and diverse motives for conducting green exercise. The sub-theme ‘individual’ is related to the contrasting motivations, durations, and frequencies of the conducted outdoor exercise. The majority of participants reported frequencies meeting the recommended physical activity guidelines, demonstrating that perhaps conducting green exercise allows individuals to meet guidelines more easily. From the collected data, adventure sport seems to last longer than other activities and this may be due to the participant being more focused on the activity than meeting certain health outcome parameters such as durations or steps.

Participant 2: “*Half an hour every day.*” [Recreational walker]

Participant 5: “*Probably minimum a session would last is about 3–4 h I would say, there’s a lot of times when you are abroad, you’re doing the activity across the course of 8–9 h of the day.*” [Snowboarder]

Participant 4: “*A rough session probably once or twice a week and I do anything between 12, 12–16 miles or so but just depending on how I feel at what time.*” [Marathon Runner]

Due to the range of the investigated outdoor activities, it was evident that motives and attitudes for green exercise are highly related to the individual and activity. The majority of the participants noted extrinsic factors for beginning to undertake their outdoor activity.

Participant 1: “*To be part of a team*” [Lacrosse Player]

Participant 2: “*Just to keep fit because I am getting a little bit older now and you need to use it or lose it.*” [Recreational walker]

Participant 3: “*Originally I am quite competitive by nature, originally I started because I wanted to start another hobby that I can continue to play into my later years, so starting now would be a good time so you can actually get better before your there.*” [Golfer]

Participant 4: “*Enjoyment would probably be the overriding one. It is probably quite good to get outside get some fresh air and see the sights, especially on a good day when suns out it is good to get out and about really.*” [Marathon runner]

#### 3.2.2. Higher Order Theme: Feelings

The higher order theme of ‘feelings’ was deduced from the predefined research question and previous studies into exercise motivations and adherence. Previous research has shown the strong connection between positive affect and feelings during and following exercise which resulted in increased motivation [36,37]. As a result, whilst the two main themes are separate, they are also strongly connected. Throughout the interviews, participants reiterated several emotional states they felt towards their outdoor activity at different time points. From the responses, it was clear that feelings, emotions, and senses during outdoor exercise fluctuate depending on what phase of the activity they are at (start, during, or end). This was evident with all participants noting different feelings dependent on what part of the activity they were discussing.

Sub-theme: Pre-activity

When questioned on feelings and emotions prior to conducting their activity, there was a large spectrum of feelings reported. Participants from recreational and competitive sport activities noted feelings such as tiredness and reductions in motivation, relating to negative feelings prior to commencing exercise. Contrastingly, adventure or recreational leisure-based activity participants responded with feelings such as excitement and eagerness prior to beginning. There was a clear dichotomy between adventure participants and recreational exercisers in terms of arousal, with recreational exercisers demonstrating a clear lack of motivation prior to beginning the activity.

Participant 1: “*Tired, just in from work, so not very motivated.*” [Lacrosse player]

Participant 2: “*Sometimes tired, sometimes good if I am getting already to go, I feel good once I get out of bed.*” [Recreational walker]

Participant 5: “*Fresh and excited I would say, and really looking forward to it.*” [Snowboarder]

Participant 3: “*I am always eager to play because you always know that you can play well, but when things start to go wrong it can quickly change, it’s one of those games golf.*” [Golfer]

#### 3.2.3. Feelings

Sub-theme: During activity

Participants from adventure sports noted that external factors such as weather and outcome performance may alter feelings during their activity. It is evident from the responses that the majority of the six participants experienced increases in positive affect and mood as a result of conducting green exercise. Feelings such as energy, focus, and positive feelings towards health were all evident during outdoor exercise. In relation to the Attention Restoration Theory [27], the majority of participants noted that mood improved following the beginning of their specific outdoor activity. The following responses highlight the effect that the powerful combination of exercise and nature can have on individuals.

Participant 1: “*Yeah, energy levels go up and concentration goes up.*” [Lacrosse player]

Participant 2: “*Good, I feel like giving myself a pat on the back, and I feel healthier I feel fitter even after the first initial part of the walk.*” [Recreational walker]

Participant 5: “*Free, I think is the best way to describe it you feel free, you feel like you are coasting and there is nothing else around you, you feel very out there, you feel free is the best way to describe it you can roam around places you normally would be able to get to.*” [Snowboarder]

Participant 6: “*Weather conditions dependent it could be pretty exhilarating, pretty tiring.*” [Sailor]

Sub-theme: Post activity

The majority of participants noted that after conducting their form of green exercise, they felt increases in positive feelings/affect and mood. In relation to previous studies, there was a clear recognition of the supplementary benefits from conducting green exercise. Whilst the majority noted they felt better in adventure sports, physical pain and reduced energy levels were evident. Furthermore, external factors in performance-based activities and sports such as the outcome of an action or the result may affect the way in which participants feel.

Participant 2: “*Yeah the endorphins make you feel happier, you feel ready to face the world, energised.*” [Recreational walker]

Participant 4: “*I’d say a good feeling after exercise obviously you got your bad feelings of being tired but it is overridden by how you feel after it.*” [Marathon runner]

Participant 5: “*Sore, normally sore, sore, tired, very very tired, you actually end up feeling hot because you’ve been cold.*” [Snowboarder]

When exploring their experiences of green exercise, many participants were in agreement that some form of physical and mental improvement occurred as a result of conducting a green exercise session. Participant’s accounts largely outlined several psychological improvements such as increased energy, concentration, happiness, and reductions in negative mood subscales.

Participant 1: “*Yeah clearer head, more focused, more energetic and happier.*” [Lacrosse player]

Participant 2: “*Well mentally because you feel sluggish and yucky, but once you’ve been out walking or running it just gets you going it makes you feel more alert and you work better.*” [Recreational walker]

Participant 3: “*Golf is quite a frustrating game so it can probably teach you quite a bit of patience.*” [Golfer]

## 4. Discussion

The research outlined in this paper aimed to understand what the primary motives are for undertaking green exercise across various forms of activities. Analysis of the full sample in the quantitative phase of the study showed that the intrinsically generated motive “*Because it makes me happy*” was the highest ranked motive for participating in green exercise. Calogiuri and Elliott (2017) [13] underlined that future studies into green exercise motivation should consider whether motives are more intrinsic or extrinsic through the use of psychological theories of motivation. In the current study, motivation was categorised through intrinsic and extrinsic motivators as in the SDT theory of human motivation [15]. With reference to this theory, based on the results of this study it may be that green exercise allows participants to develop autonomous self-regulation, which has been shown to be important in long term adherence to exercise [38]. The authors highlight that when behaviour is driven by extrinsic or controlled motivations, such as social reward or feeling forced to exercise rather than wanting to, that these are not powerful enough to sustain adherence over long periods [16]. This is somewhat reflected in the quantitative phase, with other’s opinions negatively correlating with enjoyment.

The PALMS subscales ‘enjoyment’ ‘physical condition’, and ‘psychological condition’ were ranked highest by the whole sample. This finding demonstrates that those who are currently engaged in green exercise may unconsciously or consciously understand and experience the supplementary benefits which have been shown to exist from conducting green exercise in comparison to indoor exercise [6]. This is what drives them to adhere to this form of exercise. This finding is further supported in that ‘I prefer exercising outdoors to indoors’ was ranked as the highest motive from the nature specific questionnaire. If individuals are intrinsically motivated to improve their physical or psychological health, it appears green exercise is an enjoyable way to achieve this through getting away from everyday routines. Consequently, discovering an activity that an individual enjoys and makes them feel better may be an important element in increasing the number of people using natural environments to exercise. When talking about motivation, it is also important to acknowledge barriers. Previous green exercise studies have highlighted infrastructure, weather, safety, and accessibility as barriers [8]. These barriers were also reinforced in the qualitative phase of this study. Participants from adventure sports also highlighted negative feelings in the form of soreness following their activity, which should be considered when selecting an appropriate mode of green exercise to conduct. With regards to overcoming barriers, one of the most interesting findings from the qualitative phase was that green exercise participants would rather not exercise than move to indoor environments. This underlines the powerful connection to the outdoor environment that green exercise participants have built through conducting this form of green exercise.

Previous studies have investigated motivations to conduct physical activity across various types of activities [39]. In agreement with previous studies [3,8,10], enjoyment appears to be the primary motive towards conducting green exercise for those currently engaged in this mode of exercise. This maybe unsurprising as previous research has found intrinsic motivators to be powerful in adherence to exercise [38]. A study by Roychowdhury (2012) [39] used the PALMS questionnaire to assess the motivation to conduct physical activity in club, recreational, and social participants. The study found that social level participants scored lowest on ‘mastery’ and are more motivated by external factors such as ‘affiliation’ ‘others expectations’, and ‘appearance’. In contrast to the current study, no group ranked high in any of these subscales. This perhaps highlights differences between those who conduct green exercise in comparison to those using gym-based and controlled indoor environments. The findings show that greater levels of enjoyment maybe experienced from green exercise. In terms of why this occurs, responses from the qualitative phase highlighted views and scenery, social aspects, and made indirect references to flow experiences and endorphins. This may prove an important finding in promoting green exercise to the general public and those who are currently sedentary. Research into the varying forms of green exercise has been under researched [11], where previously it may be thought that in outdoor adventure, mastery or affiliation maybe the greatest motivators [13]. The findings from the current study demonstrated that regardless of what form of green exercise is being undertaken, enjoyment remains the key determinant of adherence to exercise.

Differences between outdoor activities

The current study categorised green exercise into three levels (recreational, adventure sport, and competitive sport) which no previous study into green exercise motivation has investigated. An aim of the current study was to discover what motivational differences existed between the three forms of activities. The rank order of motivations demonstrated that all three groups put a large emphasis on enjoyment and physical condition. It was hypothesised that due to the varying costs, skills, and access to locations, differences would exist between the forms of green exercise. In agreement with the hypothesis, competitive sport participants were less motivated by the natural exercise environment. This is underlined with ‘I prefer exercising outdoors to indoors’, ‘I can experience the outdoors combined with exercise’, ‘I enjoy the views whilst exercising’, and ‘I can be in nature’ with being lowest ranked from the three groups. These results show that whilst competitive sport participants do not primarily conduct green exercise to experience the natural environment, it is perhaps the social element which evokes enjoyment. However, the exercise environment provides the area to facilitate this. Researchers Han and Wang (2018) [40] highlight that individuals who choose to shift focus away from the natural environment may not receive benefits from conducting green exercise. This may be reflected with the motivation subscale ‘psychological condition’ being ranked lowest between the three forms of green exercise within competitive sport participants. Whilst differences existed, both the recreational and adventure sport participants displayed similar responses to natural and psychological motives. As no previous study has compared differences between adventure sport and different forms of green exercise, the results demonstrate that significant differences exist compared to competitive sport participants. In fact, mastery appears to be the only motivator similar between the two forms. The results highlight that whilst each form of green exercise has distinct differences in what motivates individuals to seek out green exercise experiences, if the activity makes them feel good mentally and physically and they enjoy it then it is likely they will adhere to their selected activity.

Qualitative Findings

Previous research has examined motivations to conduct green exercise through quantitative measures. However, previous green exercise studies have underutilised mixed research methods to understand why participants seek out such environments. Furthermore, few studies have qualitatively compared differences between a range of green exercise participants. Anecdotal responses with regards to how participants felt increased mood and affect following and during a session of green exercise may be easier to interpret for the general public and may prove useful in further promotion of green exercise. The qualitative phase of the study identified two main themes from the interviews. Motivation and feelings were identified as primary themes in what drives these participants to continue to conduct and adhere to green exercise. Within these two themes, several sub-themes were developed which intermixed to tell the story of each theme. With regards to the theme ‘motivation’ there were a number of similarities in what drove participants from the different forms of green exercise to begin and continue to conduct their outdoor activity. The theme of motivation was broken down into three sub-themes: Environment, social, and individual. The participant responses revealed that these sub-themes interact to motivate participants to continue to maintain conducting green exercise. Accounts from the participants highlighted that the natural environment was an important setting, which when combined with physical activity, resulted in participants expressing increased motivation to conduct exercise.

Other interesting findings with regards to the exercise environment were related to previous developed theories regarding green exercise. The Ecological Dynamics Theory states that human behaviours, perceptions, and emotions can be shaped by the individual, task, or the environment [12]. The framework outlines how affordances offered by the exercise environment can enhance the health and wellbeing of an individual. The findings from the current study support the framework, with the majority of participants highlighting elements of the environment which increased positive feelings and motivation. Participants noted several elements related to the Ecological Dynamics Theory with temperature, control, and reduced dynamic sources of information related with indoor environments as de-motivating. Furthermore, participants highlighted they would rather not conduct exercise at all than move to an indoor environment. It appears that the reduced affordances in indoor environments reduce motivation for those already engaged in green exercise.

Previous research has also highlighted that social interaction is a reason why participants may undertake green exercise [7,41]. In agreement with these previous studies, participants cited family and friends as important factors in what motivated them to start conducting green exercise. Contrastingly, when questioned about their current exercise habits, some participants noted that they now prefer to exercise alone. This was supported by the quantitative findings in that ‘affiliation’ and ‘others expectations’ were rated as low motivators. These findings somewhat support previous research that suggests participants move down a continuum from beginning exercise via extrinsic motives and then moving towards intrinsic motives such as enjoyment and mastery [42]. This is not unusual and extrinsic motives have previously been shown to be important factors as to why individuals will begin physical activity but are a poor predictor of adherence [43].

The second main theme from the analysis was termed feelings. In agreement with previous green exercise research [3,44], all six participants noted positive changes in feelings and emotions during and after conducting their specific outdoor activity. An important part of theory-driven research was to understand how different feelings may influence an individual’s perception and motivations of green exercise. Furthermore, to investigate how this may differ at time points during an activity and the differences between these activities. Previous research has shown that positive affective responses can increase levels of motivation towards exercise [45]. This demonstrates how the two main themes are separate but linked. However, during the correlational analysis, ‘enjoyment’ and ‘psychological condition’ showed no relationship. Feelings were broken down into three sub-themes based on ‘time’ of the activity as acknowledged by previous research [3,46]. Distinct differences in feelings and emotions were evident in the analysis and therefore these three sub themes were deemed appropriate.

Vast differences in feelings, mood, and arousal before conducting the various activities were reported. As previous green exercise literature has shown, feelings, emotions, and mood will change over the course of an outdoor exercise session [46,47]. Mood has been shown to strongly influence feelings of enjoyment, stress, and quality of life [48]. Previous studies have found that changes of mood following exercise are maintained for about 2–4 h post exercise and have been found to have a positive effect on various psychological and behavioural outcome measures [46]. When investigating the motives for participation in outdoor adventure sport, it is important to acknowledge that motives may be altered during and prior to the experience [49]. Prior to conducting outdoor adventure sports, participants noted feelings such as apprehension and excitement, whereas for recreational outdoor exercise participants feelings such as tiredness and laziness were cited. These results display the large range of arousal that can occur between two differing green exercise activities and why it was deemed necessary to categorise these into three different forms. 

Public Health Recommendations

The findings of the current study demonstrate that different types of green exercise have varying motives. Individuals not currently conducting green exercise could be selected into a form based on their primary baseline motives for participation. For example, individuals that are driven by the social aspect of exercise may look to conduct outdoor competitive sports in a team or group. Similarly, those who are looking to improve their mental and physical health may opt for recreational forms of outdoor exercise such as walking clubs. Through understanding the motivations of those currently engaged in green activities these findings could be used to encourage those who have similar motivation drives but are not currently involved in any form of outdoor activity to prescribe a type of outdoor group to join. Prior to beginning to conduct green exercise, the ability to identify motives would accurately ensure appropriate activities are prescribed. The current study is in agreement with previous research which highlights that extrinsic motives are important when beginning new exercise programmes. However, in terms of adherence to exercise it appears that intrinsic motives become more important [50]. The findings are important from the perspective of public health, behaviour change, and increasing the number of available green spaces. This is one of the first studies to outline the motivational decisions and profiles between those conducting leisure time green exercise. The current study looked to build on a previous green exercise study by Calogiuri and Elliott (2017) [13] through including outdoor adventure sport as another form of green exercise. Given the rising levels of worldwide mental health issues, the findings that those currently engaged in green exercise conduct it for ‘psychological condition’ provides further evidence for green exercise as a promotional tool for preventing and managing mental health to the general public. Advocating that each form of green exercise has unique and diverse motives for conducting such exercise may allow a greater number of individuals to gain interest in beginning to conduct green exercise. Not only could this help individuals begin to conduct green exercise, but through finding an activity that meets the needs of different sub-groups of the population may be important in adherence to these forms of green exercise. Given the potential economic benefits to green exercise [7], finding methods to increase the amount individuals conducting green exercise should be a high priority. Health behaviour change has been a large problem within governments, healthcare professionals, and policy makers [51]. This study may also allow a motivational framework to be developed by such stakeholders to help promote facilitators and aid in the development of interventions to physical activity. The motivational profile of green exercise users is identified as levels of high autonomous motivation which has been shown to be important to adherence to exercise [38].

### 4.1. Limitations

As the questionnaires were self-administered online, there is a chance that the participants did not fully understand what each question was asking with no opportunity to get clarification. As a result, this may have reduced the accuracy of responses and thus reduced the reliability of the results. It may be argued whether two questionnaires are enough to sufficiently capture the complexity of an individual’s motivation. All participants were currently conducting green exercise and therefore there may be a bias towards this form of activity. However, the scope of the study was to investigate and understand the motives of those currently engaged in green exercise so as to understand what promotes adherence to such activities. Finally, there was a clear difference in the number of participants in each group which may reduce the reliability of the quantitative analysis.

### 4.2. Future Research

Following the findings of the current study, future research should seek to investigate motivations towards green exercise in various ethnic groups as previous literature has suggested that these will differ [52]. Using different measures of motivation to identify differences between the various types of green exercise is another option for future studies. Finally, as the current study did not consider gender in the quantitative phase of the study, future studies may also look to compare the differences between gender in terms of the different green exercise activities. This may also reveal important motivation differences that exist within this context as shown in previous studies.

## 5. Conclusions

The research findings detailed in this paper outlined why various forms of green exercise participants conduct green exercise. The results of the study provided significant knowledge for improving public health, through behaviour change, planning processes, and a mode of exercise which promotes long-term leisure time physical activity adherence. The findings showed that family and friends were large extrinsic motivators for individuals beginning to undertake green exercise. New participants may look to begin green exercise with friends and family to increase enjoyment as shown in previous studies [53]. This may be useful to reinforce to sedentary individuals. However, as participants continued to conduct green exercise, it appeared that intrinsic motivation, specifically enjoyment was more powerful in motivating participants to adhere to green exercise. This was the first study to compare three forms of green exercise. As hypothesised, differences in motivation between the different green exercise activities existed. Individuals who engaged in competitive sport were less motivated by the exercise environment. However, this group still rated enjoyment as the primary motive for conducting their outdoor activity. The majority of participants cited improved mood, focus, and energy after conducting their activity, suggesting that green exercise activities have the capacity to increase these various psychological subscales. The results of the research support the Ecological Dynamics framework with individuals highlighting that exercise undertaken in natural areas promotes an increase in motivation, positive behaviours, and feelings in comparison to controlled indoor settings. Based on the SDT, green exercise may increase autonomous self-regulation which promotes adherence to physical activity. As a result of the findings it is hoped that these may be applied to individuals who are currently sedentary or not involved in outdoor exercise.

## Figures and Tables

**Table 1:** Descriptive statistics for the questionnaire sample.

Group	*N*	Age (mean)	Minimum	Maximum	SD
All	182	32.2	19	68	5.85
Competitive	29	27.5	19	62	8.74
Recreational	128	38.8	20	68	12.5
Adventure	25	30.5	20	52	8.57

N—Sample number; M—Mean; SD—Standard Deviation.

**Table 2:** Rank order Physical Activity and Leisure Motivation Scale (PALMS) factors (whole sample).

Subscale	Mean	Standard Deviation
Enjoyment	4.36	0.21
Physical Condition	4.16	0.38
Psychological Condition	4.13	0.13
Mastery	3.86	0.22
Appearance	3.67	0.42
Affiliation	3.65	0.19
Competition Ego	2.43	0.18
Others Expectations	1.86	0.31

**Table 3:** Ranking of participation motives for type of green exercise.

Subscales	Rank Order	Outdoor Activity	Mean	Median
**Enjoyment**	1st	Competitive	4.57	4.24
	2nd	Recreational	4.28	4.27
	3rd	Adventure	4.28	4.60
**Physical Condition**	1st	Recreational	4.34	4.48
	2nd	Competitive	4.12	4.17
	3rd	Adventure	4.02	4.20
**Psychological Condition**	1st	Adventure	4.29	4.36
	2nd	Recreational	4.21	4.19
	3rd	Competitive	3.89	3.86
**Mastery**	1st	Competitive	4.00	4.48
	2nd	Adventure	3.94	3.88
	3rd	Recreational	3.63	3.64
**Appearance**	1st	Recreational	3.86	3.77
	2nd	Competitive	3.85	3.38
	3rd	Adventure	3.30	3.00
**Affiliation**	1st	Competitive	3.92	4.03
	2nd	Adventure	3.58	3.64
	3rd	Recreational	3.46	3.43
**Competition Ego**	1st	Competitive	3.26	3.21
	2nd	Adventure	2.10	2.04
	3rd	Recreational	1.95	1.99
**Others Expectations**	1st	Competitive	1.99	1.90
	2nd	Adventure	1.76	1.68
	3rd	Recreational	1.72	1.59

**Table 4:** Nature questionnaire for different outdoor activities.

Item	Recreational	Competitive	Adventure
I prefer exercising outdoors to indoors	4.36 **	3.83	4.65 **
I can relax from daily routines	4.46 **	4.03	4.35
I can experience the outdoors combined with exercise	4.48	3.76 **	4.54
I enjoy the views whilst exercising	4.42 **	3.24	4.69 **
I can be in nature	4.26 **	3.38	4.07 **
Exercising outdoors challenges me more	3.97	3.59	4.23
I can experience changes in nature (light, dark, sun-rain)	3.75	3.10 **	3.85
I come to places I am especially attached to	3.39 **	3.07 **	3.85
I can test new equipment and clothing	3.05	3.03	3.54

** Significant difference between 1 or more groups *p* = 0.01.

**Table 5:** PALMS subscale correlations.

Subscale								
	1	2	3	4	5	6	7	8
1	1							
2	−0.36	1						
3	0.01	−0.11	1					
4	−0.25	0.28	−0.13	1				
5	0.01	−0.47	0.24	−0.65 **	1			
6	−0.38	0.43	−0.33	−0.95	0.17	1		
7	0.15	0.61 **	0.10	0.62 **	−0.81 **	−0.15	1	
8	−0.38	−0.09	0.21	0.60 **	−0.36	0.55 *	0.19	1

1 = Appearance; 2 = Mastery; 3 = Physical condition; 4 = Affiliation; 5 = Psychological condition; 6 = Enjoyment; 7 = Competition/ego; 8= Others expectations; * *p* < 0.05; ** *p* < 0.01.

**Table 6:** Nature questionnaire item correlations.

Items	Correlations								
	1	2	3	4	5	6	7	8	9
1	1								
2	0.35 **	1							
3	0.66 **	0.43 **	1						
4	0.56 **	0.19 **	0.44 **	1					
5	0.54 **	0.17 **	0.48 **	0.54 **	1				
6	0.18 **	0.17 **	0.23 **	0.23 *	0.29 **	1			
7	0.51 **	0.31 **	0.55 **	0.40 **	0.43 **	0.26 **	1		
8	0.22 *	0.13	0.31 **	0.18	0.26 **	0.26 **	0.35 **	1	
9	0.33 **	0.15 *	0.35 **	0.21 *	0.25 **	0.01	0.35 **	0.36 **	1

1 = I can be in nature; 2 = I can relax from daily routines; 3 = I can experience the outdoors combined with exercise; 4 = I come to places I am especially attached to; 5 = I can experience changes in nature (light, dark, sun-rain); 6 = I can test new equipment and clothing; 7 = I enjoy the views whilst exercising; 8 = I prefer exercising outdoors to indoors. * *p* < 0.05; ** *p* < 0.01.

## Supplementary Materials

The following are available online at https://www.mdpi.com/1660-4601/16/10/1832/s1, Supplementary Material A: Result of motivational questionnaire; Supplementary Material B: Outdoor Exercise Interview transcriptions.
