# Supplementary material for: What Motivates Participants to Adhere to Green Exercise?
Source: Int J Environ Res Public Health. 2019 May 23;16(10):1832. doi: 10.3390/ijerph16101832 (PMC6571838; doi:10.3390/ijerph16101832)
Supplement: Supplementary file 1 [file ijerph-16-01832-s001.zip › supplementary material/Supplementary mentarial B.docx]

**Outdoor Exercise Interview transcriptions**

**Participant 1: Female, amateur Lacrosse player**

Part A: Experience

1. Describe your background of participating in your outdoor ‘activity’?
2. “I used to play at school, and I now want to get back into it.”
3. What made you start doing your outdoor ‘*activity*’?
4. “To be part of a team, to exercise outdoors with other people, to keep myself motivated and to refresh old skills.”

Part B: Activity Structure

1. Do you conduct your outdoor activity alone or in a team or group?
2. (already answered, activity is conducted in a team)

- Do you think this makes a difference to how you feel about the activity?

1. “this makes me want to do the activity more, because I don’t like exercising alone.”

- Is there anything else that being part of a team makes you want to do it?

1. “When you’re in a team you obviously don’t want to let your team mates down and this is what motivates you to do the best you can, so you bring your a-game.”
2. How long does a session last during your outdoor activity? And how often do you conduct this?
3. “An hour and a half once a week, and we have games on a Saturday”

- And how long would a game last?

1. Games last around an hour to an hour and a half.

Part C: Location

1. Describe the location where your activity is conducted?
2. “On old hockey pitches outside”

- So is it grass or synthetic pitches?

1. “Astroturf”
2. If given the option would you rather exercise outdoors than indoors? And if so why?
3. “Outdoors, because you’ve got the fresh air as well, and yeah.”

- Would you say this makes you feel better?

1. “Yeah”

- Do you prefer exercise in a greenspace as opposed to a man-made environment?

1. “greenspace – field”

Part D: Barriers to activity

1. What barriers do you feel exist to conducting your (activity)? Or what prevents you doing it?
2. “Either injuries or time constraints”

- Would you say you don’t have enough time through work?

1. “Yeah”

- Does the season of your affect participation rates in your activity?

1. “yeah if it’s raining people will more likely not want to turn up”

- Does temperatures effect participation? Does training get cancelled or is this in the off season in the winter?

1. This is the offseason

- How do you overcome these barriers

1. “Train indoors”

- In terms of time, do you just make time to conduct the activity in your leisure time?

1. “Yeah”

Part E: Feelings and emotions

1. Before conducting your outdoor (*activity*) how do you feel?
2. “Tired, just in from work, so not very motivated”
3. Whilst conducting your outdoor (*activity*) how does it make you feel?
4. “Yeah, energy levels go up and concentration goes up.”
5. After conducting your outdoor (*activity*) how does it make you feel?
6. “Great” > do you feel a lot better? “Yeah a lot better”
7. Overall do you think conducting your activity benefits you physically and/or mentally?
8. “Both, especially mentally” > What physical differences do you think exist? “Stronger, more cardio which is better for running around” > Mentally? “Yeah clearer head, more focused, more energetic and happier.”

Part F: Motivations

1. What is the primary reason you exercise you currently conduct your activity?
2. “To be part of a team”
3. What elements of the environment motivate you to conduct your activity (or do they?)
4. “Just being outdoors”
5. Do you ever feel distracted by the outdoor environment?
6. “no”
7. What features of the natural environment you exercise in do you frequently notice (*give examples*)?
8. “If the sun is shining down pretty hard and you can’t see and like look where you are it kind of distracts you from what you’re supposed to be doing.”

“I’d like to thank you for participating in the interview, if you have any further questions you can ask them now, > de-brief form”

**Participant 2: Female, recreational walker**

Part A: Experience

1. Can you describe your background of participating in your activity?
2. “Yeah I started probably about ten years ago, prior to that I was jogging but now that I’ve got older I prefer walking.”
3. What made you start doing your (*activity*)?
4. “For health, to lose weight and for the feel good factor” > Could you describe what you mean by feel good factor? > “Just makes you feel good, when you come back you want to eat healthier, you feel more motivated, you feel like you can take on the world, you feel better with yourself.”

Part B: Activity Structure

1. Do you conduct your outdoor activity alone or in a team or group?
2. “Alone.”

- Do you think this makes a difference to how you feel about the activity? Would you feel different in a group? Or do you prefer doing it alone?

1. “I prefer doing it alone.” > Any reasons why? > “Just because you can sort of lose yourself and you can go at your own pace and you can go where you want and you can just please yourself.”
2. How long does a session last during your outdoor activity? And how often do you conduct this?
3. “Half an hour every day and yeah.”

Part C: Location

1. Describe the location where you most often conduct your activity?
2. “Emm, it’s called Clunny Hill, the woods, the park.” > So it’s almost a sort of public park would you say? “Yeah, it’s also got woods.”
3. If given the option would you rather exercise outdoors than indoors? And some reasons perhaps why?
4. “Outdoors, because indoors it is hot and sweaty and there’s people around you and it is just nicer outside, you feel better.”

- Do you prefer exercise in a greenspace as opposed to a man-made environment? For example, rather exercise in a park as opposed to the streets?

1. “Yes” > Any reasons why again? “Just because you feel better, it is less noisy, you know you just, its just nicer and it is healthier, I don’t know it’s more fresh air.”

Part D: Barriers to activity

1. What barriers do you feel exist to conducting your (activity)? Or what prevents you doing this?
2. “Sometimes laziness, not so much the weather but that can play a part if it is icy for example, injury and just maybe motivation if you stop it is hard to get started again.”

- You may have addressed this already but does the season of your affect participation rates in your activity?

1. “Yeah, probably from April to October is the best, March to October.” And that would be due to? > “Light, it is much better for you, I don’t feel it is the same when it is dark.”

- How do you overcome these barriers (So you listed some barriers to exercise such as motivation, darkness, so how do you overcome these barriers?)

1. “I just have to sort of get up and go for it, once you get started you go for it, emm if I put on weight that is another motivation and getting older you know you need to go out walking to keep yourself going.”

Part E: Feelings and emotions

1. Before conducting your outdoor (*activity*) how do you feel?
2. “Sometimes tired, sometimes good if I am getting already to go, I feel good once I get out of bed.” So you would say that you most often conduct this activity in the morning then? “Yeah.”
3. Whilst conducting your outdoor (*activity*) how does it make you feel?
4. “Good, I feel like giving myself a pat on the back, and I feel healthier I feel fitter even after the first initial part of the walk.”
5. After conducting your outdoor (*activity*) how does it make you feel?
6. “Even better, because I’ve done.” >so you would say it increases the way you feel after exercise? “Yeah the endorphins make you feel happier, you feel ready to face the world, energised.”
7. Overall do you think conducting your activity benefits you physically and/or mentally?
8. “Both.” > Can you go into a little more detail about either? “Well mentally because you feel sluggish and yucky, but once you’ve been out walking or running it just gets you going it makes you feel more alert and you work better, you eat better, what was the other part of the question?” > Any physical benefits you feel you gain from walking? “Well obviously you feel leaner, even if you, I don’t know, you can lose a bit of weight and even if you don’t your body feels like its reacting in a good way to exercise.”

Part F: Motivations

1. What is the primary reason you exercise you currently conduct your activity?
2. “Just to keep fit because I am getting a little bit older now and you need to use it or lose it and a bit of weight or but I don’t believe that.” >So you would say that health benefits are the primary reason? “yeah”
3. What elements of the environment motivate you to conduct your activity (or do they?) So does the actual environment where you conduct it, drive you to exercise?
4. “Yeah, I suppose when it is nicer weather especially first thing in the morning when the sun comes up and it is a nice location, you know you want to get out and do it.”
5. Do you ever feel distracted by the outdoor environment?
6. “Not really I do take in bits and bobs, but you tend to lose yourself more than noticing what’s going on” > So your more thinking about your day, rather than what is going on around you? “Yeah but you can notice the changes in the season and things like that.”
7. What features of the natural environment you exercise in do you frequently notice (*give examples*)? So you’ve noted things like the change in light and others
8. “The changing leaves, the leaves. Every April/May they are budding and at the end of the year they are all starting to wilt.”

“I’d like to thank you for participating in the interview, if you have any further questions you can ask them now”

**Participant 3: Recreational Golfer**

Part A: Experience

1. Can you describe your background of participating in your activity?
2. “I started playing golf, maybe about 6-7 years ago now, I got into it because I was a bit older and it was a more sociable game and a lot of my friends were playing it, so I decided to start taking it up.”
3. What made you start doing your (*activity*)?
4. (already addressed in previous question) So that’s what you’d say made you take it up? “Yeah”

Part B: Activity Structure

1. Do you conduct your outdoor activity alone or in a team or group?
2. “Emm, a bit of a mixture really, I go out on my own to practice but most of the time it is in a group of two or three folk and we’ll go out and play bounce games most of the time not too many competitions, but I’m wanting to enter more competitions.” > So at the moment your quite recreational would you say? “Yeah”

- Do you think this makes a difference to how you feel about the activity?

1. “Probably depends, when I’m on my own I’m not so annoyed if I am playing badly, if I’m in a group and I don’t play well it’s more annoying and frustrating when your playing badly with other folk. However if you play well with other people it is a lot better than when playing on your own and you play well.”
2. How long does a session last during your outdoor activity? And how often do you conduct this?
3. “Well when I am at home I try to get out at least a couple times a week, and I’d probably say an average round is probably only going to be about 3 hours depending on who you are playing in front of or behind.”

Part C: Location

1. Describe the location where your activity is conducted?
2. “Most of the time it is Forres golf club, but I work with boys that live all over Scotland so try to play different course when I can, when other people are available but mainly Forres but occasionally different places as well.
3. If given the option would you rather exercise outdoors than indoors? So a specific example for golf would be would you rather exercise on the course or a simulator indoors?
4. “Definitely outdoors during the summer when the weather is good I prefer to be outside, however in the winter golf is not much of an enjoyable sport to be outside for three to potentially four hours when it is raining for example, or really cold simulators and things can be good through the winter but yeah generally it is a better weather sport.”

- Do you prefer exercise in a greenspace as opposed to a man-made environment? N/A for golf

Part D: Barriers to activity

1. What barriers do you feel exist to conducting your (activity)?
2. “Just like I just said. Weather I am less likely to play when the weather is bad, and if I am feeling sore, if I’ve been to the gym and doing anything around the house which has been quite strenuous it puts you off playing because you know you will be stiff and won’t play as well because of that.”

- Does the season of your affect participation rates in your activity?

1. “Yes” As previously answered

- How do you overcome these barriers, such as the season or feeling sore

1. “Well the weather is one that you can’t really do much about, I do tend to go out even if it is cold as long as it is not raining too much, because if you don’t play during the winter Summer is usually quite hard.”

Part E: Feelings and emotions

1. Before conducting your outdoor (*activity*) how do you feel?
2. “I am always eager to play because you always know that you can play well, but when things start to go wrong it can quickly change, it’s one of those games golf. Your mood changes a lot during the 18 holes.”
3. Whilst conducting your outdoor (*activity*) how does it make you feel? So you would perhaps say mixed emotions based on your last response?
4. “Yeah mixed emotions depending on performance.”
5. After conducting your outdoor (*activity*) how does it make you feel?
6. “Depends, yeah depends on the way you play.”
7. Overall do you think conducting your activity benefits you physically and/or mentally?
8. “Emm, yeah I think is not too strenuous physically a lot of older guys obviously play so for someone younger than myself it is not too strenuous. But yeah it is good to get out and get regular exercise.” > Do you think there are any mental benefits from golf? “Probably, I think mentally golf is quite a frustrating game so it can probably teach you quite a bit of patience, emm yeah I think it can benefit you quite a lot.”

Part F: Motivations

1. What is the primary reason you exercise you currently conduct your activity?
2. “Originally I am quite competitive by nature, originally I started because I wanted to start another hobby that I can continue to play into my later years, so starting now would be a good time so you can actually get better before your there. And things like football and other activities are harder to come by when I work away so coming home it is quite hard to then get in a team or consistently play a game like that.” > So basically you’d say to improve yourself? “yeah”
3. What elements of the environment (golf course) motivate you to conduct your activity (or do they?)
4. “Yeah when it is good weather it is nice to play a nice course. Yeah.”
5. Do you ever feel distracted by the outdoor environment?
6. “They don’t distract me, but they are nice to take it.” >So they can grab your attention but they don’t distract you from your game? “yeah”
7. What features of the natural environment you exercise in do you frequently notice (*give examples*)?
8. “Uphills”

“I’d like to thank you for participating in the interview, if you have any further questions you can ask them now, > de-brief form”

**Participant 4: Marathon Runner**

Part A: Experience

1. Describe your background of participating in your outdoor ‘activity’?
2. “So basically my background is I’ve run one marathon, going to be running number two coming in May, I have not trained as much for this one as I had for the last one. The last marathon I trained since, for about 6 months prior to the start, starting it. Trained maybe once or twice a week, distances, up to, from 12-18 miles or kilometres depending on what I was choosing to run on the night, it was more outside based work.”
3. What made you start doing your (*activity*)?
4. “It was more of a fun element, to get outdoors a bit more, something to set myself a challenge and something to work towards but more fun than competition running.”

Part B: Activity Structure

1. Do you conduct your outdoor activity alone or in a team or group?
2. “Alone as I am not part of any running group.”

- Do you think this makes a difference to how you feel about the activity?

1. “Individually I think it is a lot easier for me, I work a lot better individually, I don’t know if it would alter my performance if it was done in a team but individually is what I choose to do.”
2. How long does a session last during your outdoor activity? And how often do you conduct this?
3. “A rough session probably once or twice a week and I do anything between 12, 12-16 miles or so but just depending on how I feel at what time.”

Part C: Location

1. Describe the location where your activity is conducted?
2. “Well I live in the city so most of my running is occurs starting in the middle of the city and working my way outside the city, so there is sort of canal roots, more out towards the countryside where it’s easier and more free to run.”
3. If given the option would you rather exercise outdoors than indoors?
4. “Definitely outdoors, I don’t really see the interest in running indoors, it is a lack of motivation wise, at least when you are running outside there are more things to see, more things to watch and kind of distract you more than when you’re inside it is more a case of just running for the sake of running on the same spot. But yeah definitely more to explore than anything else.”

- Do you prefer exercise in a greenspace as opposed to a man-made environment?

1. “Well yeah definitely a greenspace I obviously start in the city, so it is a lot more difficult when you are in the city, you’ve got different obstacles you’ve got to move around, which is sometimes good and it takes your mind off actually running. But so does when you’re actually going out to the countryside, you get to see more different views, different scenery. You get to see the environment and the different environmental factors really.”

Part D: Barriers to activity

1. What barriers do you feel exist to conducting your (activity)?
2. “External barriers or internal barriers?” > Either, just what would stop you doing your activity? > “Majorly would be injury, injury is quite a hard one for myself, other than that, possibly weather difficulties obviously with the winter it is hard to get your running in during the snow periods that might turn me away from running outside, but it would be that much of a down turn if it was bad weather other than snow really.”

- Does the season of your affect participation rates in your activity?

1. “Yeah only in the extreme seasons.”

- How do you overcome these barriers?

1. “Seasonal I probably just wouldn’t train that much, I wouldn’t be more inclined to train inside due to bad weather, I’d just leave it and wait for another day. In terms of injury it is more of a case of just letting it run out, maybe slight jogs and things like that but there is not really much I can do about the injuries.”

Part E: Feelings and emotions

1. Before conducting your outdoor (*activity*) how do you feel?
2. “Running is more of a thing I like to do when I am feeling good, I’d say like I feel I get a lot more out of my running when I am feeling up for a run, rather than if I was feeling I didn’t want to run I wouldn’t run but if I was feeling good before a run, I’d probably run for quite a long distance.”
3. Whilst conducting your outdoor (*activity*) how does it make you feel?
4. “Good, emm as I say I only run when I’m up to it, so when I’m actually out on the track or whatever I am running around. It’s a lot more easier to run so it is a good feeling kind of just like taking in what I see really.”
5. After conducting your outdoor (*activity*) how does it make you feel?
6. “Obviously good, good that you’ve actually done some exercise that you’ve got out and about, beats kind of just lumbering about for a bit but yeah I’d say a good feeling after exercise obviously you got your bad feelings of being tired but it is overridden by how you feel after it.”
7. Overall do you think conducting your activity benefits you physically and/or mentally?
8. “Yes to both.” > Could you say why? > “Well physically is an obvious one, if you are training for a marathon your going to want to make yourself do exercise to better your end goal of your time and stuff like that. Mentally it is more about a good feeling, I do it when I am running it does expand all my happiness levels but as I say when I go out running I am happy anyway.”

Part F: Motivations

1. What is the primary reason you exercise you currently conduct your activity?
2. “Enjoyment would probably be the overriding one. It is probably quite good to get outside get some fresh air and see the sights, especially on a good day when suns out it is good to get out and about really.”
3. What elements of the environment motivate you to conduct your activity (or do they?)
4. “Covered”
5. Do you ever feel distracted by the outdoor environment?
6. “Always yes,”
7. What features of the natural environment you exercise in do you frequently notice (*give examples*)?
8. “Living in Edinburgh, on the outskirts there is a lot of good things to see such as Authors Seat and you’ve got Portobello down by the beach. So a lot of different environmental areas where you can see and take in really.”

“I’d like to thank you for participating in the interview, if you have any further questions you can ask them now, > de-brief form”

**Participant 5: Snowboarder**

Part A: Experience

1. Can you describe your background of participating in your activity?
2. “I skied a lot when I was younger and then got into snowboarding about four years ago. The experience with skiing such as falling over and getting cold and hurt helped with the fact when you are snowboarding and you fall over and get cold and hurt as well but the psychological side is what puts a lot of people off but that helped a lot. I’ve been doing it for four years solid now, been abroad every single year doing it so that helps. Also the fact is that it is on our doorstep helps even more.
3. What made you start doing your (*activity*)?
4. “What made me start doing it was as I said previously the fact that I skied before and my friends did that so snowboarding seemed a lot more fun to do and looked more enjoyable and I like the fact that you are out in the fresh air. In the winter you can’t really go up for a game of golf as much or play football as much so it keeps you active and basically gets you outdoors and you can see different parts of the world that not many others get to see doing the activity.”

Part B: Activity Structure

1. Do you conduct your outdoor activity alone or in a team or group?
2. “I’ve only ever been up once and done it myself. It’s something that I see as needing two people to do it just makes it a bit more enjoyable. But yes you can go up on your own, but it just makes it more enjoyable if there is two or three or four or five or ten it doesn’t really matter it is an activity that can be done.”

- Do you think this makes a difference to how you feel about the activity?

1. “Yeah, alone it is not as much fun I would say as you can’t really laugh at anybody else when they are falling over or hurting themselves. Your more inclined.. you don’t stop as much when you are on your own, you just keep going and going but when you are with other people, you wait for each other, you watch each other, you have a laugh and roll about. So yeah I do see it a bit differently actually.”
2. How long does a session last during your outdoor activity? And how often do you conduct this?
3. “Probably minimum a session would last is about 3-4 hours I would say, there’s a lot of times when you are abroad you’re doing the activity across the course of 8-9 hours of the day but 2-3 hours of that you are resting or stopping for lunch or a drink but I would say you are doing at least a minimum of four hours a time when you are doing it.”

Part C: Location

1. Describe the location where your activity is conducted?
2. “The activity is conducted on a hill, normally at altitudes far higher than wherever at and you have to get up there via lift and its normally got to be cold for it to snow.”
3. If given the option would you rather exercise outdoors than indoors? (outdoors on the mountain or indoors on an artificial dry ski slope)
4. “Outdoors, one of the main reasons I enjoy doing the activity is purely for the fresh air and getting out and about, and the views and seeing things is the best part about it.”

- Do you prefer exercise in a greenspace as opposed to a man-made environment?

1. N/A for this activity.

Part D: Barriers to activity

1. What barriers do you feel exist to conducting your (activity)?
2. “The barriers is obviously the weather. If it is too windy or you can’t see visibility wise, then you can’t do it and it is not enjoyable. And also you need snow so it has to be different times of the month, different times of the year. So you need snow for it so that is a barrier it is all weather based.”

- Does the season of your affect participation rates in your activity?

1. “Yes, 100%”

- How do you overcome these barriers

1. “Not so much without using artificial methods such as going down to Glasgow, it is something I have never done, I don’t see the idea of going inside to snowboard or artificial nowhere near the same aspect of doing it in real life. I would rather pay the money and get abroad and do it at times of the year when you can’t do it back home.”

Part E: Feelings and emotions

1. Before conducting your outdoor (*activity*) how do you feel?
2. “Fresh and excited I would say, and really looking forward to it.”
3. Whilst conducting your outdoor (*activity*) how does it make you feel?
4. “Free, I think is the best way to describe it you feel free, you feel like you are coasting and there is nothing else around you, you feel very out there, you feel free is the best way to describe it you can roam around places you normally would be able to get to.”
5. After conducting your outdoor (*activity*) how does it make you feel?
6. “Sore, normally sore, sore, tired, very very tired, you actually end up feeling hot because you’ve been cold, so whenever you go anywhere hot you feel very red, very windchilled, very hot, but the thing about it is that even when you wake up the next day and you are sore, it is the kind of activity you just rub some deep heat on you and when you get going again after twenty minutes, your legs and body start to loosen up again.”
7. Overall do you think conducting your activity benefits you physically and/or mentally?
8. “Yes, 100%, physically and mentally because it is very physically demanding on you on your body as well as getting through the psychological part of hurting yourself and getting back up and mentally yes it is because it gets you out in the fresh air and it gets you doing things that you don’t think you’d be able to do.”

Part F: Motivations

1. What is the primary reason you exercise you currently conduct your activity?
2. “Probably about making me feel good and making me feel at that time of year when you can’t really get as much out of other activities outdoors, it is probably the most active activity outdoors you can probably do, especially between the months of the year that is available.”
3. What elements of the environment motivate you to conduct your activity (or do they?)
4. “Yeah 100% if you go over to places such as France or Switzerland or Italy and Austria if you see the views that I have seen and the pictures that I have taken will never ever do it justice, it is something that I think everyone should experience in their life it is something that I am quite glad I managed to do. Sometimes twice a year for the last 3-4 year because I enjoy it that much.”
5. Do you ever feel distracted by the outdoor environment?
6. “Yeah definitely I think it grabs your attention more than anything else, you forget where you are half the time.”
7. What features of the natural environment you exercise in do you frequently notice (*give examples*)?
8. AS ABOVE.

“I’d like to thank you for participating in the interview, if you have any further questions you can ask them now, > de-brief form”

**Participant 6: Sailor**

Part A: Experience

1. Can you describe your background of participating in your activity?
2. **“**Well, I suppose I started sailing when I was about, I don’t know... 6,7,8 I don’t know. I’ve mainly sailed at club level but sailed in the Scottish championships, the national championships and the U.K.”
3. What made you start doing your (*activity*)?
4. “I think probably started doing it because I stayed in Findhorn, which is next to the sea and everybody sailed and my family sailed so I took up sailing at an early age.”

Part B: Activity Structure

1. Do you conduct your outdoor activity alone or in a team or group?
2. “I suppose when I started it, it was probably in a boat of 2 or 3 but we used to go to the royal Findhorn yacht club cadets and that was a group for training. At the moment I am sailing in a single handed boat.”

- Do you think this makes a difference to how you feel about the activity?

1. “Suppose it does a bit, but then you can’t blame anyone else for your mistakes so but and the good thing about doing it on your own is that you don’t have to rely on anyone to crew with you and crews are quite hard to get so you can just sail when you want.”
2. How long does a session last during your outdoor activity? And how often do you conduct this?
3. “A normal race would take about an hour to an hour and a half, and it is work dependent I suppose. When I was younger I sailed probably every Saturday and Sunday in the Summertime.

Part C: Location

1. Describe the location where your activity is conducted? In your activities case this would just be the sea then?
2. “Well Findhorn Bay is an inland bay but you can sail on the sea as well yeah.”
3. Perhaps not really related to your activity however, If given the option would you rather exercise outdoors than indoors?
4. “Definitely outdoors,” >Any reasons particularly why? > “Just feels better.”

- Do you prefer exercise in a greenspace as opposed to a man-made environment?

1. NOT APPLICABLE TO SPORT

Part D: Barriers to activity

1. What barriers do you feel exist to conducting your (activity)?
2. “Suppose the main barriers in Findhorn are the tidal situation. So you can only sail when the tide is in. Other barriers are probably work related.”

- Does the season of your affect participation rates in your activity?

1. “Yes.”

- How do you overcome these barriers

1. “Retire and sail more.”

Part E: Feelings and emotions

1. Before conducting your outdoor (*activity*) how do you feel?
2. “Depending on the weather conditions I could be apprehensive, could be worried about the weather if it is quite windy but yeah.”
3. Whilst conducting your outdoor (*activity*) how does it make you feel?
4. “Again weather conditions dependent it could be pretty exhilarating, pretty tiring.”
5. After conducting your outdoor (*activity*) how does it make you feel?
6. “Good, yeah can’t think too much.”
7. Overall do you think conducting your activity benefits you physically and/or mentally?
8. “Definitely yes.”

Part F: Motivations

1. What is the primary reason you exercise you currently conduct your activity?
2. “Keeping fit and being involved in sport”
3. What elements of the environment motivate you to conduct your activity (or do they?)
4. “I suppose you could say that Findhorn Bay is safe to sail.”
5. Do you ever feel distracted by the outdoor environment?
6. “I suppose Findhorn Bay is a lovely area to sail in but I am probably more concentrated on the sailing yeah.”
7. What features of the natural environment you exercise in do you frequently notice (*give examples*)?
8. “I don’t know, I suppose there is the Culbin forest so you looking at that quite a lot.”

“I’d like to thank you for participating in the interview, if you have any further questions you can ask them now, > de-brief form”
